# Prognostic Impact of the Hevylite Assay in Patients With IgG or IgA Multiple Myeloma Treated Within the GMMG‐MM5 Trial

**DOI:** 10.1111/ejh.70061

**Published:** 2025-11-11

**Authors:** Tim Richardson, Elias Mai, Ekaterina Menis, Axel Benner, Diana Tichy, Kaya Miah, Mathias Hänel, Britta Besemer, Amelie Boquoi, Igor Wolfgang Blau, Christian S. Michel, Hans Walter Lindemann, Snjezana Janjetovic, Peter Brossart, Helga Bernhard, Peter Reimer, Hans Salwender, Dirk Hose, Anja Seckinger, Marc Raab, Hartmut Goldschmidt, Christof Scheid, For the German‐Speaking Myeloma Multicenter Group (GMMG) HD5 Investigators, Koordinierungszentrum für Klinische Studien, KKS) Heidelberg, Facharztpraxis Hämatologie und Onkologie, Praxisnetzwerk Hämatologie/Internistische Onkologie, Facharztpraxis Onkologie, Caritas Krankenhaus Bad Mergentheim, MVZ Baden‐Baden, Charité Universitätsmedizin Berlin, Charité Universitätsmedizin Berlin, HELIOS Klinikum Berlin‐Buch, Medizinisches Versorgungszentrum, Facharztpraxis Onkologie, Onkologie Seestrasse, Klinikum Bielefeld Mitte, Facharztpraxis Onkologie, Universitätsklinikum Bonn, Johanniter Krankenhaus Bonn, Onkologie Rheinsieg, Städt. Klinikum Braunschweig, Onkologische Schwerpunktpraxis, Onkologische Praxis im Krankenhaus Buchholz, Onkologische Schwerpunktpraxis, Klinikum Chemnitz, Regiomed Kliniken GmbH, Carl‐Thiem‐Klinikum Cottbus, Klinikum Darmstadt, Onkologische Schwerpunktpraxis, Gemeinschaftspraxis für Hämatologie und Onkologie, Fachpraxis für Hämatologie und Onkologie, Universitätsklinikum Essen, Evangelisches Krankenhaus Essen‐Werden Zentrum für Innere Medizin, Universitätsklinikum Frankfurt, Agaplesion Medizinisches Versorgungszentrum, MVZ) Frankfurt, Krankenhaus Nordwest, Interdisziplinäres Facharztzentrum, IFS) Frankfurt, Vitanus GmbH, Frankfurter Rotkreuz‐Kliniken, PIOH‐Praxis für Internistische Onkologie und Hämatologie, Onkologische Facharztpraxis, Facharztpraxis für Hämatologie und Onkologie, Kath. Krankenhaus Hagen, Asklepios Klinik Altona, Asklepios Klinik St. Georg, Hämatologisch‐Onkologische Praxis Altona, Facharztpraxis für Hämatologie und Onkologie, OncoResearch Lerchenfeld UG, Evangelisches Krankenhaus Hamm gGmbH, Onkologische Schwerpunktpraxis, Klinikum Hanau GmbH, Klinikum Region Hannover, Onkologisches Ambulanzzentrum OAZ Hannover, Universitätsklinikum Heidelberg, Onkologische Schwerpunktpraxis, Onkologische Schwerpunktpraxis Heilbronn, SLK Kliniken Heilbronn GmbH, Facharztpraxis für Hämatologie und Tumorerkrankungen, Universitätsklinikum des Saarlandes, Klinikum Idar‐Oberstein, Schwerpunktpraxis für Hämatologie und Onkologie, Gemeinschaftspraxis für Hämatologie, Universitätsklinikum Köln, Onkologie Köln, Praxis Internistischer Onkologie und Hämatologie, Kliniken Köln, Facharztpraxis für Hämatologie, Onkologisches Zentrum Lebach, Klinikum der Stadt Ludwigshafen am Rhein, Onkologische Schwerpunktpraxis Lüneburg, Universitätsmedizin der Johannes Gutenberg‐Universität Mainz, MED Facharztzentrum, Universitätsmedizin Mannheim, Mannheimer Onkologie Praxis, Facharztpraxis für Innere Medizin, Praxis für Innere Medizin, AöR) Johannes Wesling Klinikum Minden, Kliniken Maria Hilf, Städtisches Klinikum München, Facharztpraxis für Innere Medizin, medius Kliniken, Onkologische Facharztpraxis, Paracelsus Kliniken, medius Kliniken, Medizinisches Versorgungszentrum am Siloah St. Trudbert‐Klinikum, Onkologische Praxis Pinneberg, Gemeinschaftspraxis Innere Medizin/Onkologie, Facharztpraxis für Onkologie, Diakonie‐Klinikum Schwäbisch Hall, Zaho‐Zentrum für ambulante Hämatologie und Onkologie, Diakonie Klinikum Jung‐Stilling, Gastroenterologie Onkologie Bodensee, Onkologische Schwerpunktpraxis Speyer, Marienhospital Stuttgart, Krankenhaus der Barmherzigen Brüder Trier, Klinikum Mutterhaus der Borromäerinnen Trier, Onkologische Schwerpunktpraxis am Brüderkrankenhaus Trier, Onkologie Rheinsieg, Universität Tübingen, OMM Optimed Mundial GmbH, Facharztpraxis für Onkologie, Ammerland Klinik, Facharztpraxis für Onkologie

**Affiliations:** ^1^ Department of Internal Medicine I University Hospital Cologne Cologne Germany; ^2^ Heidelberg Myeloma Center, Internal Medicine V, Hematology, Oncology and Rheumatology Heidelberg University Hospital Heidelberg Germany; ^3^ Division of Biostatistics German Cancer Research Center (DKFZ) Heidelberg Germany; ^4^ Department of Internal Medicine III Chemnitz Germany; ^5^ Department of Hematology, Oncology and Immunology University Hospital Tübingen Tübingen Germany; ^6^ Department of Hematology University Clinic Essen Essen Germany; ^7^ Medical Clinic Charité University Medicine Berlin Berlin Germany; ^8^ Department of Internal Medicine III University Medical Center Mainz Mainz Germany; ^9^ Department of Hematology and Oncology Hagen Germany; ^10^ Department of Hematology and Cell Therapy Helios Klinikum Berlin‐Buch Berlin Germany; ^11^ Department of Internal Medicine, Oncology, Hematology, Cell‐ and Immunotherapies, Clinical Immunology and Rheumatology University Hospital Bonn Bonn Germany; ^12^ Internal MedicineV Darmstadt Germany; ^13^ Kliniken Essen‐Mitte, Klinik für Hämatologie, Internistische Onkologie Und Stammzelltransplantation Pattbergstr Essen Germany; ^14^ Asklepios Tumorzentrum Hamburg Hamburg Germany; ^15^ Laboratory of Hematology and Immunology & Labor für Myelomforschung Vrije Universiteit Brussel Jette Belgium; ^16^ Internal Medicine V, Hematology, Oncology and Rheumatology GMMG Study Group at the Heidelberg University Hospital Heidelberg Germany

**Keywords:** heavylite assay, multiple myeloma, prognostic biomarker

## Abstract

Response assessment during treatment of multiple myeloma (MM) typically relies on immunofixation and serum electrophoresis. However, low levels of IgG and especially IgA paraprotein are difficult to quantify reliably. The Hevylite Assay quantifies the kappa and lambda fractions of IgG and IgA separately and is useful to determine response to therapy. Using serum samples of 360 evaluable patients from the prospective GMMG‐MM5 trial (EudraCT‐No. 2010–019173‐16) we assessed the normalization of the kappa/lambda ratio with the Hevylite Assay (HLCr) at baseline, after induction, mobilization, autologous blood stem cell transplantation, consolidation and every three months during maintenance or follow‐up within two years after the end of consolidation. We observed a steady increase in the proportion of patients with normalized HLCr over the course of therapyAchieving HLCr normalization any time until the end of consolidation was associated with a trend towards a prolonged progression‐free survival (PFS; hazard ratio (HR) = 0.75, 95% confidence interval (95% CI) = 0.56–1.01, *p* = 0.06) but not overall survival (OS; HR = 0.94, 95% CI = 0.69–1.26, *p* = 0.66) in multivariable time‐dependent Cox regression analyses. Using a landmark analysis from the end of consolidation there was again a marginally statistically significant effect of HLCr normalization by the end of consolidation on PFS using a multivariable Cox model on the subset of the two study arms with continuous lenalidomide maintenance (HR 0.61, 95% CI 0.37–1.02, *p* = 0.06). No such effect was observed in study arms in which maintenance was only applied to patients not in CR at the end of consolidation.

In conclusion, our analysis of the Hevylite Assay in patients with IgG or IgA myeloma from the GMMG‐MM5 study did not find evidence to support the general use of HLCr normalization as a response parameter for predicting PFS or OS. However, the differential effects of HLCr normalization depending on the way in which treatment was adapted to response may be of interest for future study designs on response‐adapted therapy.

**Trial Registration:** ISRCTN05622749.

## 1 Introduction

The treatment of multiple myeloma (MM) has undergone fundamental changes in the last decades, moving from chemotherapy to immunomodulatory drugs (IMIDs), proteasome inhibitors and antibodies to T‐cell engaging immunotherapies [1]. Using these new therapies with increasing potential to induce rapid and deep remissions has prompted the development of novel ways to assess the tumor burden, in particular methods to detect minimal residual disease (MRD) in the bone marrow by either next generation flow or next generation sequencing [2]. While these techniques are indispensable tools for clinical studies, the need for bone marrow puncture and the complex and costly laboratory analyses have so far limited their broad use in clinical routine worldwide. Therefore, it would be desirable to use minimally invasive blood sampling to assess tumor activity and a more sensitive assessment of circulating paraprotein might be an appealing candidate for this task.

The monoclonal protein may be a complete immunoglobulin composed of two heavy chains and two light chains or, in cases of light chain MM, only light chains. The production of light chains exceeds that of heavy chains [3]. This imbalance results in an elevated level of light chains in the serum, which can also appear in the urine once renal reabsorption capacity is exceeded [4]. In addition to serum protein electrophoresis (SPE) and immunofixation electrophoresis (IFE) the assessment of free light chains (FLC) in peripheral blood is widely used to monitor myeloma burden and the normalization of the kappa/lambda ratio (FLCr) is part of the definition of stringent complete response (CR) [5]. The prognostic effect of normalization of the FLCr on progression‐free survival (PFS) and overall survival (OS) has been shown [6, 7]. In accompanying research to the GMMG‐MM5 trial (EudraCT‐No. 2010–019173‐16), a statistically significant prognostic effect of FLCr has been shown as well [8].

In 2009, The Binding Site Ltd. (Birmingham, United Kingdom) introduced a novel immunoassay called the Hevylite Assay to precisely measure both the involved and uninvolved immunoglobulin isotypes. This test employs sheep polyclonal antibodies targeting junctional epitopes in the constant region between the heavy and light chains (heavy/light chain, HLC). These HLC antibodies specifically identify different immunoglobulin classes, such as IgGκ, IgGλ, IgAκ, IgAλ, IgMκ, and IgMλ. Measurements are made in pairs (e.g., IgGκ/IgGλ) to determine the ratios of involved to uninvolved immunoglobulins, similar to the FLC κ/λ ratios [9].

Research using the HLC assay suggests that HLC ratios (HLCr) are valuable for screening, monitoring, and risk stratifying patients with multiple myeloma (MM), Amyloid Light‐chain (AL) amyloidosis, Waldenström's macroglobulinemia, and monoclonal gammopathy of undetermined significance (MGUS) [7, 10, 11, 12]. In addition, HLC testing can reflect restoration of plasma cell homeostasis, i.e., recovery of normal plasma cell populations, which has been shown in several studies to correlate with improved progression‐free and overall survival [13, 14]. Furthermore, in some cases, these ratios may serve as sensitive markers of disease progression [13]. The utility of the HLC assay to identify and quantify the paraprotein even when SPE and IFE are difficult to evaluate, has been shown [15, 16].

Thus, in a trial with a response‐adapted treatment design, we aimed to investigate whether a more detailed analysis of paraprotein response using HLCr normalization would provide additional prognostic information on clinical outcomes focusing on patients with IgG or IgA myeloma.

## 2 Methods

### 2.1 GMMG‐MM5 Trial

Transplant‐eligible patients with newly diagnosed MM and measurable disease were enrolled in the prospective, multicenter, phase III MM5 trial (EudraCT No. 2010–019173‐16) and 601 patients were randomly assigned to one of four treatment arms. Details regarding eligibility criteria, study design, and primary endpoints have been published elsewhere [17]. Following randomization, participants underwent induction therapy with three cycles of either bortezomib, doxorubicin, and dexamethasone (PAd) or bortezomib, cyclophosphamide, and dexamethasone (VCD). Stem cell mobilization, high‐dose melphalan therapy, and autologous stem cell transplantation (ASCT) were performed in accordance with local protocols. After these procedures, 2 cycles of lenalidomide and dexamethasone were administered for consolidation, followed by lenalidomide maintenance therapy continuously for two years in the two arms PAd‐CR and VCD‐CR, while in the other two arms PAd‐noCR and VCD‐noCR it was given to patients not in CR and until CR was achieved (Figure S1; Supporting Information). The GMMG‐MM5 trial received approval from the ethics committees of all participating institutions, with the University of Heidelberg serving as the lead ethics committee (AFmu‐119/2010). All participants provided written informed consent.

### 2.2 Assessment of Paraprotein

Using the commercially available Hevylite assay (The Binding Site, Birmingham, UK), kappa and lambda fractions of the IgG or IgA paraprotein were measured on serum samples from baseline, the end of induction, after high‐dose therapy, at the end of consolidation and during the maintenance phase. Normal ranges have been established by the manufacturer and are as follows: IgGκ 3.84–12.07 g/L; IgGλ 1.91–6.74 g/L; IgGκ/IgGλ 1.12–3.21; IgAk 0.57–2.08 g/L; IgAλ 0.44–2.04 g/L; IgAk/IgAλ 0.78–1.94. For free light chains, the manufacturer's reference range of κ/λ ratio 0.26–1.65 was applied. A HLCr within the reference range was recorded as normal [13].

### 2.3 Statistical Methods

During the course of therapy until the end of consolidation, FLCr and HLCr were coded as normal or abnormal if they were inside or outside the reference range. FLCr and HLCr were used as time‐dependent covariates in a multivariable Cox regression analysis for PFS and OS, respectively. HLCr normalization was defined as the presence of a normal HLCr during therapy until the end of consolidation. Response at the end of consolidation (CR versus no CR) was used as an additional time‐dependent covariate. Age (years), International Staging System (ISS, stages I, II and III), high‐risk cytogenetics (yes, no; defined as either deletion 17p13 and/or translocation t(4;14) and/or gain 1q21 (> 3 copies)), paraprotein type (IgA or IgG) and study arm (PAd‐CR vs. VCD‐CR vs. PAd‐noCR vs. VCD‐noCR) were included as fixed covariates. Results were reported using hazard ratio estimates and corresponding 95% confidence intervals (CI). The proportional hazards assumption was checked graphically using scaled Schoenfeld residuals.

The curves for PFS and OS and the corresponding 95% CIs were derived using the method of Kaplan and Meier. Landmark Cox regression analyses were performed from the end of consolidation to assess the impact of HLCr normalization at this time point. No multiplicity adjustments were performed for exploratory analyses. Values of *p* < 0.05 were considered statistically significant. The analyses were conducted using R version 4.3.2 (https://www.R‐project.org, Vienna, Austria).

## 3 Results

The expanded population of the GMMG‐MM5 trial consisted of 604 patients (ref. https://doi.org/10.1038/s41375‐020‐0976‐9). Three patients were excluded due to violation of inclusion criteria. Of the 601 patients included in the intention to treat (ITT) population, 360 had an IgA or IgG paraprotein and available serum samples. Besides the type of paraprotein there was no difference in baseline characteristics between these patients and the remaining 241 patients of the ITT population (Table 1).

**TABLE 1:** This table shows no imbalances between the two subsets except for the myeloma subtype.

	Evaluable	Not evaluable	Overall
	(*N* = 360)	(*N* = 241)	(*N* = 601)
Age			
Mean (CV%)	58.1 (14.3%)	58.1 (13.4%)	58.1 (13.9%)
Median [Q1, Q3]	59.0 [52.0, 65.0]	59.0 [53.0, 64.0]	59.0 [53.0, 65.0]
SEX			
Male	217 (60.3%)	135 (56.0%)	352 (58.6%)
Female	143 (39.7%)	106 (44.0%)	249 (41.4%)
WEIGHT			
Mean (CV%)	79.6 (19.8%)	77.7 (20.2%)	78.8 (20.0%)
Median [Q1, Q3]	79.0 [68.0, 90.0]	75.0 [66.0, 87.0]	78.0 [68.0, 89.0]
Beta2 microglobuline			
Mean (CV%)	5.10 (128.9%)	7.09 (166.3%)	5.90 (153.9%)
Median [Q1, Q3]	3.40 [2.40, 5.13]	3.80 [2.63, 7.70]	3.50 [2.50, 5.90]
Albumin			
Mean (CV%)	36.9 (17.3%)	39.4 (18.0%)	37.9 (17.9%)
Median [Q1, Q3]	37.3 [32.3, 41.4]	40.4 [34.9, 44.9]	38.2 [33.0, 43.0]
Missing	1 (0.3%)	4 (1.7%)	5 (0.8%)
Renal impairment^a^			
Yes	29 (8.1%)	54 (22.4%)	83 (13.8%)
No	331 (91.9%)	187 (77.6%)	518 (86.2%)
WHO score			
0	155 (43.1%)	97 (40.2%)	252 (41.9%)
1	170 (47.2%)	115 (47.7%)	285 (47.4%)
2	26 (7.2%)	21 (8.7%)	47 (7.8%)
3	5 (1.4%)	5 (2.1%)	10 (1.7%)
Not known	4 (1.1%)	3 (1.2%)	7 (1.2%)
Mprotein heavy chain, number of patients (%)			
IgA	99 (27.5%)	24 (10.0%)	123 (20.5%)
IgG	261 (72.5%)	103 (42.7%)	364 (60.6%)
IgD	0 (0%)	9 (3.7%)	9 (1.5%)
Light chain myeloma	0 (0%)	105 (43.6%)	105 (17.5%)
Mprotein light chain			
Kappa, mg/L	249 (69.2%)	155 (64.3%)	404 (67.2%)
Lambda, mg/L	111 (30.8%)	86 (35.7%)	197 (32.8%)

*Note:* Values for paraprotein subtype are given as number of patients (percentage).

^a^ Creatinine > 2 mg/dL and/or glomerular filtration rate < 40 mL/min.

Both groups were also comparable in terms of response at the end of consolidation therapy when considering the 4 different arms of the study. The proportion of patients in CR after consolidation varied between 8.9% and 26.5% in the evaluable cohort and between 19.7% and 28.8% in the remaining patients (Table S1).

**FIGURE 1:**
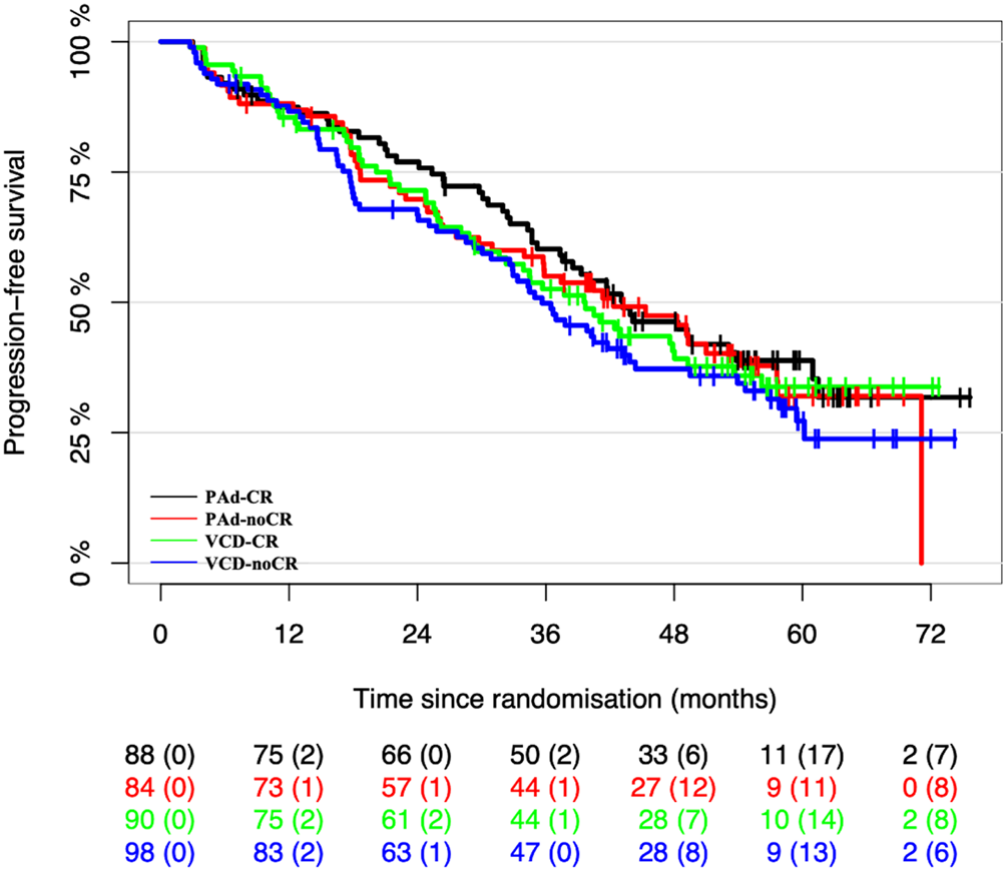
With a median follow‐up of 57, 6 months, 222 PFS events were observed in 360 evaluable patients. The distribution of PFS for the subset of the Hevylite analysis, is calculated by the method of Kaplan and Meier, stratified by randomization arm.

Figure 1 shows the PFS curves of the four study arms.

At first, we aimed to replicate the findings by Klein et al. [7] on the prognostic impact of FLCr normalization in patients with MM treated within the GMMG‐MM5 trial when selecting only patients with immunoglobulin paraprotein.

When assessing the normalization of FLCr as a time‐dependent covariate in a multivariable Cox regression, it was found to be a statistically significant prognostic factor for PFS (HR 0.71, 95% CI 0.51–1.00, *p* = 0.05) and marginally statistically significant for OS (HR 0.67, 95% CI 0.43–1.05, *p* = 0.08), confirming the role of FLC ratio normalization in a cohort of patients with only IgG or IgA paraprotein.

When analysing the effect of normalization of HLCr using a corresponding multivariable Cox regression, there was a trend towards an improved PFS (Table 2, HR 0.75, 95% CI 0.56–1.01, *p* = 0.06), while high‐risk cytogenetics and ISS were found to be the strongest prognostic factors for PFS and OS. Normalization of HLCr showed no clear effect (HR 0.94, 95% CI 0.69–1.26, *p* = 0.66) in multivariable time‐dependent Cox regression analysis with respect to overall survival (Table 3).

**TABLE 2:** Multivariable Cox regression to evaluate the prognostic impact of HLCr normalization on PFS (A).

Covariate	Covariate levels	Hazard ratio	95% CI	*p*
Study arm	PAd‐CR	Ref		
	PAd‐noCR	0.96	[0.63;1.47]	0.85
	VCD‐CR	1.09	[0.73;1.64]	0.67
	VCD‐noCR	1.16	[0.77;1.75]	0.47
Hlc.norm.	No	Ref		
	Yes	0.75	[0.56;1.01]	0.06
Response cons	CR	Ref		
	No CR	0.92	[0.60;1.40]	0.68
High risk	No	Ref		
	Yes	1.62	[1.20;2.19]	0.002
ISS	I	Ref		
	II	1.36	[0.98;1.90]	0.07
	III	2.14	[1.47;3.11]	< 0.001
Age		1.00	[0.98;1.02]	0.97
Mprotein.subtype	IgA	Ref		
	IgG	0.98	[0.69;1.39]	0.92

**TABLE 3:** Multivariable Cox regression to evaluate the prognostic impact of HLCr normalization on OS (B).

Covariate	Covariate levels	Hazard ratio	95% CI	*p*
Study arm	PAd‐CR	Ref		
	PAd‐noCR	1.19	[0.77;1.82]	0.43
	VCD‐CR	1.34	[0.88;2.04]	0.18
	VCD‐noCR	1.39	[0.91;2.14]	0.13
Hlc.norm.	No	Ref		
	Yes	0.94	[0.69;1.26]	0.66
Response cons	CR	Ref		
	No CR	1.21	[0.80;1.81]	0.37
High risk	No	Ref		
	Yes	1.52	[1.09;2.14]	0.01
ISS	I	Ref		
	II	1.45	[1.05;2.00]	0.02
	III	2.64	[1.78;3.93]	< 0.001
Age		1.01	[0.99;1.02]	0.58
Mprotein.subtype	IgA	Ref		
	IgG	0.88	[0.62;1.23]	0.45

In addition, a multivariable Cox regression landmark analysis for PFS and OS from the end of consolidation was performed. Again, high‐risk cytogenetics and ISS were found to be statistically significant prognostic factors both for PFS and OS, while HLCr normalization showed a trend for better PFS (HR 0.76, 95% CI 0.53–1.09, *p* = 0.13; Table 4) but not for OS (HR 0.90, 95% CI 0.49–1.65, *p* = 0.73; Table 5).

**TABLE 4:** Landmark analysis from the end of consolidation to evaluate the prognostic impact of HLCr normalization on PFS (A).

Covariate	Covariate levels	Missing	Hazard ratio	95% CI	*p*
HLCr norm.	No	0	Ref		
	Yes		0.76	[0.53;1.09]	0.13
Study arm	PAd/VCD‐CR	0	Ref		
	PAd/VCD‐noCR		1.01	[0.71;1.43]	0.96
High risk	No	34	Ref		
	Yes		1.78	[1.23;2.55]	0.002
ISS	I	0	Ref		
	II		1.29	[0.86;1.94]	0.22
	III		2.01	[1.29;3.15]	0.002
Age		0	0.99	[0.97;1.01]	0.29

**TABLE 5:** Landmark analysis from the end of consolidation to evaluate the prognostic impact of HLCr normalization on OS (B).

Covariate	Covariate levels	Missing	Hazard ratio	95% CI	*p*
HLCr norm.	No	0	Ref		
	Yes		0.90	[0.49;1.65]	0.73
Study arm	PAd/VCD‐CR	0	Ref		
	PAd/VCD‐noCR		1.86	[1.04;3.33]	0.04
High risk	No	34	Ref		
	Yes		3.04	[1.72;5.35]	< 0.001
ISS	I	0	Ref		
	II		1.29	[0.62;2.70]	0.50
	III		3.97	[1.97;8.03]	< 0.001
Age		0	1.01	[0.98;1.05]	0.53

**FIGURE 2:**
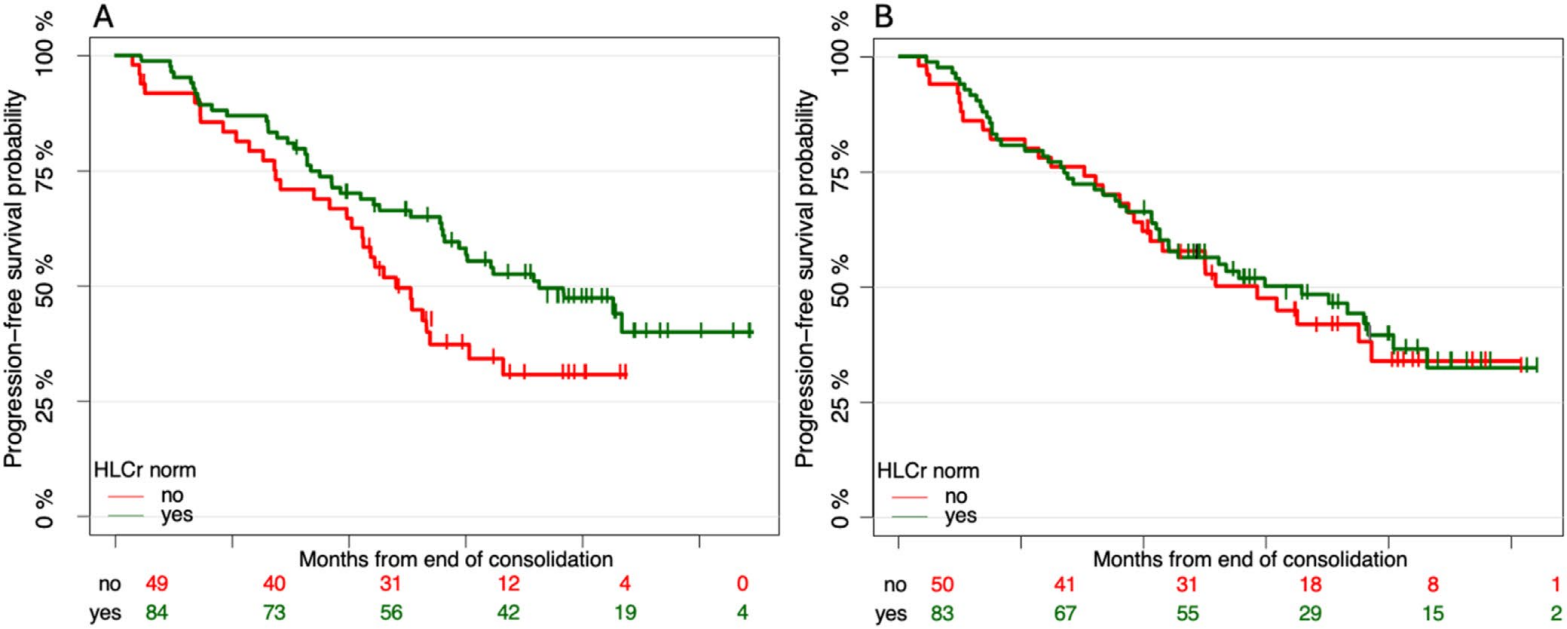
Progression‐free survival from end of consolidation stratified by normalization of HLCr in arms PAd‐CR/VCD‐CR (A) and in arms PAd‐noCR/VCD‐noCR (B).

Since the GMMG‐MM5 trial investigated two different maintenance strategies, the analysis was repeated separately for the continuous (PAd‐CR and VCD‐CR) and response‐adapted maintenance arms (PAd‐noCR and VCD‐noCR) of the study. For patients receiving lenalidomide for 2 years regardless of response, HLCr normalization was found to be marginally statistically significantly associated with better PFS in a multivariable Cox regression landmark analysis from the end of consolidation (HR 0.61, 95% CI 0.37–1.02, *p* = 0.06; Table S2). In contrast, no effect of HLC normalization on PFS was seen in patients receiving lenalidomide only when not achieving a CR after consolidation in multivariable Cox landmark analysis, as shown in Figure 2 (Table S3, HR 0.86, 95% CI 0.51–1.45, *p* = 0.58).

## 4 Discussion

With increasingly effective treatment options in MM, response assessment must be improved to increase the sensitivity to detect lower disease burden. For IgG or IgA MM, immunofixation is still the gold standard [18], however there are often borderline results reported with the detection of faint bands of paraprotein. In these cases, the interpretation will be dependent on the investigator to be classified as positive or negative. Measuring total IgG or IgA may also be misleading since at diagnosis there is often an increase of the pathological Ig with a simultaneous depression of the corresponding polyclonal, normal Ig. In patients responding to therapy this is reversed and while the monoclonal Ig is reduced there may be recovery of the polyclonal Ig and thus the total level of IgG or IgA may not anymore reflect tumor burden.

It therefore seems attractive to separately assess the kappa and lambda portions of IgG or IgA and be able to calculate the ratio, similarly to the free light chain kappa/lambda ratio. This is the basis for the Hevylite assay, quantifying IgA or IgG in terms of kappa or lambda HLC usage. A study in 156 MM patients with IgG or IgA paraprotein showed a significantly better OS when responding patients also had a normal HLCr at the time of best response [13]. In a prospective study comparing autologous to allogeneic SCT in MM patients, normalization of all HLCr (IgG, IgA and IgM) before transplant was significantly related to PFS and OS in a multivariate analysis [19]. In the IFM2009 study HLC response was used to further stratify patients with either CR or VGPR after consolidation [14]. PFS in this analysis was significantly better when HLC response also was classified as CR based on the difference between the lambda and kappa HLC results.

With these heterogeneous results regarding the added value of HLC testing, we used the data from the prospective GMMG‐MM5 trial and performed HLC analyses on serum samples at different time points during treatment in the subgroup of patients with either IgG or IgA paraprotein. Using the normalization of the HLC kappa/lambda ratio as a time‐dependent covariate, we could not demonstrate a significant correlation with PFS or OS. In contrast the normalization of the FLC ratio showed a significant effect on PFS and OS, thus confirming our previous results for the total GMMG‐MM5 cohort in a subgroup of patients with only IgG or IgA paraprotein. We speculated that the longer half‐life of intact immunoglobulins may have caused these divergent effects and analyzed the impact of HLCr normalization at the end of consolidation therapy by landmark analysis. For the total study cohort there was no statistically significant impact of HLCr normalization on either PFS or OS. However, when separating the patients according to the two different maintenance strategies, there was a significant impact of HLCr normalization on PFS in patients randomized to continuous lenalidomide maintenance, while no such effect was found in the study arms with response‐adapted maintenance. This aligns with the notion that HLCr normalization may be more informative among patients who achieve deep responses such as CR, by further discriminating those with favorable outcomes. In contrast, in patients with less than CR, other adverse prognostic features may drive outcomes more strongly than HLC results.

This observation underscores that the prognostic significance of a given treatment response may vary considerably depending on the subsequent therapeutic strategy. Our results indicate that patients achieving an optimal HLC response derive the greatest benefit from continued lenalidomide maintenance. One possible explanation is that normalization of the HLC ratio reflects recovery of normal humoral immunity—analogous to polyclonal reconstitution observed on serum electrophoresis—which may enhance the immunomodulatory effects of lenalidomide in controlling multiple myeloma. Conversely, in patients who discontinued lenalidomide maintenance despite achieving complete remission (approximately 25% of patients in arms PAd‐noCR and VCD‐noCR), those with HLCr normalization may have received less cumulative lenalidomide exposure compared to patients in arms PAd‐CR and VCD‐CR. Meanwhile, patients without HLCr normalization in the same arms could have benefited from longer maintenance therapy, potentially offsetting the impact of a less favorable response through more intensive treatment.

However, while the Hevylite assay is providing separate quantification of the kappa and lambda portions of IgG or IgA paraproteins and despite the theoretical benefits of this approach, our data do not support its general use to assess treatment response to predict PFS or OS. While there may be reasons to use the Hevylite test in special situations in MM, better options for sensitive paraprotein quantification such as mass spectrometry (MS) are needed. MS has emerged as a more sensitive alternative, capable of detecting low levels of monoclonal proteins in peripheral blood. In the GMMG‐MM5 trial, MS proved feasible for response monitoring [20]. Kubicki et al. demonstrated that MS negativity post‐transplant correlates with longer PFS [21] while Puig et al. found that MS outperformed immunofixation in sensitivity and risk stratification [22]. Additionally, Lasa et al. reported that peripheral residual disease detected by next‐generation flow cytometry is associated with a significantly higher risk of progression or death, underscoring the potential of less invasive monitoring methods [23]. Another approach to quantify low levels of disease but avoiding the necessity to perform bone marrow aspiration is the analysis of cell‐free DNA from peripheral blood [24]. Nevertheless, Hevylite may still hold value in prospective studies by further stratifying outcomes within patients achieving deeper responses, even though this role is likely to be increasingly superseded by more sensitive MS‐ and NGS‐based MRD assays.

One caveat of our approach may be the way in which the HLC results were analyzed. While it appeared plausible to use the normalization of the kappa/lambda ratio similarly to FLC, other analyses may have yielded other results. One such example would be the use of FLC after CART therapy, where suppression of both kappa and lambda FLC—rather than FLC ratio normalization—was predictive of PFS and OS [25].

In summary our analysis of Hevylite assay results in patients with IgG or IgA MM from the GMMG‐MM5 study does not support the general use of HLC ratio normalization with the Hevylite assay as a response parameter for assessing the course of disease. However, the strikingly different results in relation to the different maintenance strategies may be of interest for the discussion of suitable response endpoints (e.g., MRD) to trigger response‐adapted treatment strategies.

## Author Contributions

Conceptualization: Hartmut Goldschmidt, Marc Raab, Christof Scheid, Elias Mai, Hans Salwender. Methodology, formal analysis: Axel Benner, Diana Tichy, Kaya Miah. Patient recruiting: Helga Bernhard, Mathias Hänel, Igor‐Wolfgang Blau, Britta Besemer, Snjezana Janjetovic, Peter Brossart, Amelie Boquoi, Christian S. Michel, Hans Walter Lindemann, Peter Reimer, Hans Salwender, Dirk Hose, Anja Seckinger. Original draft preparation: Christof Scheid and Tim Richardson. Supervision: Hartmut Goldschmidt. Project administration: Ekaterina Menis. All authors have read and agreed to the published version of the manuscript.

## Ethics Statement

The study was conducted according to the guidelines of the Declaration of Helsinki and approved by the local ethics committees of all participating centers (leading ethics committee University of Heidelberg AFmu‐119/2010).

## Consent

Informed consent was obtained from all subjects involved in the study.

## Conflicts of Interest

The authors declare no conflicts of interest.

## Supporting information


**Data S1:** ejh70061‐sup‐0001‐supinfo.docx.

## Data Availability

Data from published parts of the GMMG‐MM5 trial can be made available upon request to and decision of the principal investigator (Hartmut Goldschmidt; hartmut.goldschmidt@med.uni-heidelberg.de) and the board of directors of the GMMG.

## Appendix A

The GMMG thanks the Koordinierungszentrum für Klinische Studien (KKS) Heidelberg for the support of the trial and data monitoring. The GMMG thanks all participating investigators and centers: Facharztpraxis Hämatologie und Onkologie, Aschaffenburg; Praxisnetzwerk Hämatologie/Internistische Onkologie, Bad Honnef; Facharztpraxis Onkologie, Bad Kreuznach; Caritas Krankenhaus Bad Mergentheim, Medizinische Klinik 2, Bad Mergentheim; MVZ Baden‐Baden, Medizinisches Versorgungszentrum des Klinikums Mittelbaden, Baden‐Baden; Charité Universitätsmedizin Berlin, Campus Benjamin Franklin, III. Medizinische Abteilung, Berlin; Charité Universitätsmedizin Berlin, Charité Campus Mitte, Medizinische Klinik mit Schwerpunkt Onkologie und Hämatologie, Berlin; HELIOS Klinikum Berlin‐Buch, Klinik für Hämatologie, Onkologie und Immunologie, Berlin; Medizinisches Versorgungszentrum (MVZ), Onkol. Schwerpunkt am Oskar‐Helene‐Heim, Berlin; Facharztpraxis Onkologie, Gastroenterologie, Hämatologie, Palliativmedizin, Berlin; Onkologie Seestrasse, Berlin; Klinikum Bielefeld Mitte, Klinik für Hämatologie, Onkologie und Palliativmedizin, Bielefeld; Facharztpraxis Onkologie, Gastroenterologie, Hämostaseologie, Palliativmedizin, Bochum; Universitätsklinikum Bonn, Med. Klinik und Poliklinik III, Schwerpunkte Onkologie, Hämatologie und Rheumatologie, Bonn; Zaho–Bonn, Zentrum für ambulante Hämatologie und Onkologie, Bonn; Johanniter Krankenhaus Bonn, Internistische Onkologie, Bonn; Onkologie Rheinsieg, Praxisnetzwerk Hämatologie/Internistische Onkologie, Bonn‐Beuel; Städt. Klinikum Braunschweig, Medizinische Klinik III, Braunschweig; Onkologische Schwerpunktpraxis, Braunschweig; Onkologische Praxis im Krankenhaus Buchholz, Buchholz; Onkologische Schwerpunktpraxis, Celle; Klinikum Chemnitz, Innere Medizin III, Chemnitz; Regiomed Kliniken GmbH, Medizinisches Versorgungszentrum (MVZ) Coburg des Klinikums Coburg, Coburg; Carl‐Thiem‐Klinikum Cottbus, II. Medizinische Klinik, Cottbus; Klinikum Darmstadt, Medizinische Klinik V Hämatologie/Onkologie, Darmstadt; Onkologische Schwerpunktpraxis, Darmstadt; Gemeinschaftspraxis für Hämatologie und Onkologie, Medizinisches Zentrum am St.‐Josefs‐Hospital, Dortmund; Fachpraxis für Hämatologie und Onkologie, Erfurt; Universitätsklinikum Essen, Klinik für Hämatologie, Essen; Evangelisches Krankenhaus Essen‐Werden Zentrum für Innere Medizin, Klinik für Hämatologie, Onkologie und Stammzelltransplantation, Essen; St. Antonius‐Hospital, Klinik für Hämatologie und Onkologie, Eschweiler; Universitätsklinikum Frankfurt, Goethe‐Universität, Medizinische Klinik II, Hämatologie, Onkologie, Rheumatologie, Infektiologie, Frankfurt am Main; Agaplesion Medizinisches Versorgungszentrum (MVZ) Frankfurt, Frankfurt am Main; Krankenhaus Nordwest, Klinik für Onkologie und Hämatologie, Frankfurt am Main; Interdisziplinäres Facharztzentrum (IFS) Frankfurt, Ambulantes Krebszentrum (AKS), Frankfurt am Main; Vitanus GmbH, Frankfurt am Main; Frankfurter Rotkreuz‐Kliniken, Klinik Maingau, Abt. Hämatologie/Onkologie und Palliativmedizin, Frankfurt am Main; PIOH‐Praxis für Internistische Onkologie und Hämatologie, Frechen; Onkologische Facharztpraxis, Gerlingen; Facharztpraxis für Hämatologie und Onkologie, Gießen; Kath. Krankenhaus Hagen, St.‐Josefs‐Hospital, Klinik für Hämatologie und Onkologie, Hagen; Asklepios Klinik Altona, Abteilung Onkologie mit Sektion Hämatologie, Hamburg; Asklepios Klinik St. Georg, Abteilung Hämatologie, Onkologie und Stammzelltransplantation, Hamburg; Hämatologisch‐Onkologische Praxis Altona (HOPA), Hamburg; Facharztpraxis für Hämatologie und Onkologie, Hamburg; OncoResearch Lerchenfeld UG, Hamburg; Evangelisches Krankenhaus Hamm gGmbH, Medizinische Klinik, Hämatologie/Onkologie, Hamm; Onkologische Schwerpunktpraxis, Hanau; Klinikum Hanau GmbH, Medizinische Klinik III, Hanau; Klinikum Region Hannover, Klinikum Siloah, Onkologie und Palliativmedizin, Hannover; Onkologisches Ambulanzzentrum OAZ Hannover, Hannover; Universitätsklinikum Heidelberg, Medizinische Klinik V, Heidelberg; Onkologische Schwerpunktpraxis, Heidelberg; Onkologische Schwerpunktpraxis Heilbronn, Heilbronn; SLK Kliniken Heilbronn GmbH, Medizinische Klinik III, Heilbronn; Facharztpraxis für Hämatologie und Tumorerkrankungen, Henningsdorf; Universitätsklinikum des Saarlandes, Innere Medizin I, Homburg/Saar; Klinikum Idar‐Oberstein, Medizinische Klinik I, Idar‐Oberstein; Westpfalz‐Klinikum, INN1, Kaiserslautern; Schwerpunktpraxis für Hämatologie und Onkologie, Kaiserslautern; Gemeinschaftspraxis für Hämatologie, Onkologie und Infektiologie, Karlsruhe; Institut für Versorgungsforschung in der Onkologie (InVo), Koblenz; Universitätsklinikum Köln, Klinik I Innere Medizin, Köln; Onkologie Köln, Gemeinschaftspraxis für Onkologie und Hämatologie, Köln; Praxis Internistischer Onkologie und Hämatologie (PIOH), Köln; Kliniken Köln, Krankenhaus Köln‐Holweide, Köln; Facharztpraxis für Hämatologie, Onkologie und Gerinnung, Kronach; Onkologisches Zentrum Lebach, Caritaskrankenhaus Lebach, Lebach; Klinikum der Stadt Ludwigshafen am Rhein, Medizinische Klinik A, Ludwigshafen a. Rh.; Onkologische Schwerpunktpraxis Lüneburg, Lüneburg; Universitätsmedizin der Johannes Gutenberg‐Universität Mainz, III. Medizinische Klinik, Mainz; MED Facharztzentrum, Gemeinschaftspraxis für Hämatologie und Onkologie, Mainz; Universitätsmedizin Mannheim, III. Medizinische Klinik, Hämatologie und Internistische Onkologie, Mannheim; Mannheimer Onkologie Praxis, Mannheim; Facharztpraxis für Innere Medizin, Hämatologie und Onkologie, Mannheim; Praxis für Innere Medizin, Hämatologie und internistische Onkologie, Marburg; Mühlenkreiskliniken (AöR) Johannes Wesling Klinikum Minden, Hämatologie/Onkologie, Hämostaseologie und Palliativmedizin, Minden; Kliniken Maria Hilf, Krankenhaus St. Franziskus, Klinik für Hämatologie, Onkologie und Gastroenterologie, Mönchengladbach; Städtisches Klinikum München, Klinikum Harlaching, Klinik für Hämatologie, Onkologie und Palliativmedizin, München; Facharztpraxis für Innere Medizin, Hämatologie und Onkologie, Neunkirchen; medius Kliniken, Klinik Nürtingen, Klinik für Innere Medizin Onkologie, Hämatologie, Nürtingen; Onkologische Facharztpraxis, Oberhausen; Paracelsus Kliniken, Klinik Osnabrück, Innere Medizin/Hämatologie und Onkologie, Osnabrück; medius Kliniken, Klinik Ostfildern‐Ruit, Innere Medizin, Gastroenterologie und Tumormedizin, Ostfildern; Medizinisches Versorgungszentrum am Siloah St. Trudbert‐Klinikum, Pforzheim; Onkologische Praxis Pinneberg, Pinneberg; Gemeinschaftspraxis Innere Medizin/Onkologie, Pirmasens; Facharztpraxis für Onkologie, Rosenheim; Diakonie‐Klinikum Schwäbisch Hall, Klinik für Innere Medizin III, Schwäbisch Hall; Zaho‐Zentrum für ambulante Hämatologie und Onkologie, Siegburg; Diakonie Klinikum Jung‐Stilling, Innere Medizin, Siegen; Gastroenterologie Onkologie Bodensee, Praxis Singen, Singen; Onkologische Schwerpunktpraxis Speyer, Speyer; Marienhospital Stuttgart, Zentrum für Innere Medizin III, Onkologie, Hämatologie, Palliativmedizin, Stuttgart; Krankenhaus der Barmherzigen Brüder Trier, Innere Medizin I, Trier; Klinikum Mutterhaus der Borromäerinnen Trier, Innere Medizin I, Trier; Onkologische Schwerpunktpraxis am Brüderkrankenhaus Trier, Praxis für Innere Medizin, Nephrologie, Hämatologie und Onkologie, Trier; Onkologie Rheinsieg, Praxisnetzwerk Hämatologie und Internistische Onkologie, Troisdorf; Universität Tübingen, Medizinische Klinik, Abtl. II/Hämatologie, Onkologie, Immunologie und Rheumatologie, Tübingen; OMM Optimed Mundial GmbH, Viersen; Facharztpraxis für Onkologie, Wendlingen/Esslingen; Ammerland Klinik, Medizinische Klinik, Westerstede; Facharztpraxis für Onkologie, Westerstede; Onkologische Schwerpunktpraxis, Wolfsburg–Helmstedt; Praxis Wolfsburg, Wolfsburg.
