# Supplementary material for: Prognostic Impact of the Hevylite Assay in Patients With IgG or IgA Multiple Myeloma Treated Within the GMMG‐MM5 Trial
Source: Eur J Haematol. 2025 Nov 11;116(3):226–34. doi: 10.1111/ejh.70061 (PMC12861712; doi:10.1111/ejh.70061)
Supplement: Supplementary file 1 — Data S1: ejh70061‐sup‐0001‐supinfo.docx. [file EJH-116-226-s001.docx]

**Prognostic Impact of the Hevylite™ Assay in Patients with IgG or IgA Multiple Myeloma treated within the GMMG-MM5 Trial**

Tim Richardson^1^, Elias Mai^2^, Ekaterina Menis^2^, Axel Benner^3^, Diana Tichy^3^, Kaya Miah^3^, Mathias Hänel^4^, Britta Besemer^5^, Amelie Boquoi^6^, Igor Wolfgang Blau^7^, Christian S Michel^8^, Hans Walter Lindemann^9^, Snjezana Janjetovic^10^, Peter Brossart^11^, Helga Bernhard^12^, Peter Reimer^13^, Hans Salwender^14^, Dirk Hose^15^, Anja Seckinger^15^, Marc Raab^2^, Hartmut Goldschmidt^16^, Christof Scheid^1^ for the German-Speaking Myeloma Multicenter Group (GMMG) HD5 investigators^17^

1. Department of Internal Medicine I, University Hospital Cologne, 50937 Cologne, Germany
2. Heidelberg Myeloma Center, Internal Medicine V, Hematology, Oncology and Rheumatology, Heidelberg University Hospital, Heidelberg, Germany
3. Division of Biostatistics, German Cancer Research Center (DKFZ), (69120) Heidelberg, Germany
4. Department of Internal Medicine III, Klinikum Chemnitz, 09116 Chemnitz, Germany
5. Department of Hematology, Oncology and Immunology, University Hospital Tübingen, 72076 Tübingen, Germany
6. Department of Hematology, University Clinic Essen, 45147 Essen, Germany
7. Medical Clinic, Charité University Medicine Berlin, 13353 Berlin, Germany
8. Department of Internal Medicine III, University Medical Center Mainz, 55131 Mainz, Germany
9. Department of Hematology and Oncology, Katholisches Krankenhaus Hagen, 58097 Hagen, Germany
10. Department of Hematology and Cell Therapy, Helios Klinikum Berlin-Buch, Berlin, Germany.
11. Department of Internal Medicine, Oncology, Hematology, Cell- and Immunotherapies, Clinical Immunology and Rheumatology, University Hospital Bonn, 53127 Bonn, Germany
12. Internal Medicine V, Klinikum Darmstadt, 64283 Darmstadt, Germany
13. Kliniken Essen-Mitte, Klinik für Hämatologie, Internistische Onkologie und Stammzelltransplantation Pattbergstr. 1-3, 45239 Essen, Essen, Deutschland
14. Asklepios Tumorzentrum Hamburg, AK Altona and AK St. Georg, 22763 Hamburg, Germany
15. Laboratory of Hematology and Immunology & Labor für Myelomforschung, Vrije Universiteit Brussel, Laarbeeklaan 103, 1090, Jette, Belgium
16. Internal Medicine V, Hematology, Oncology and Rheumatology, GMMG Study Group at the Heidelberg University Hospital, Heidelberg, Germany
17. Investigators are shown in Appendix 1

Supplementary Materials:


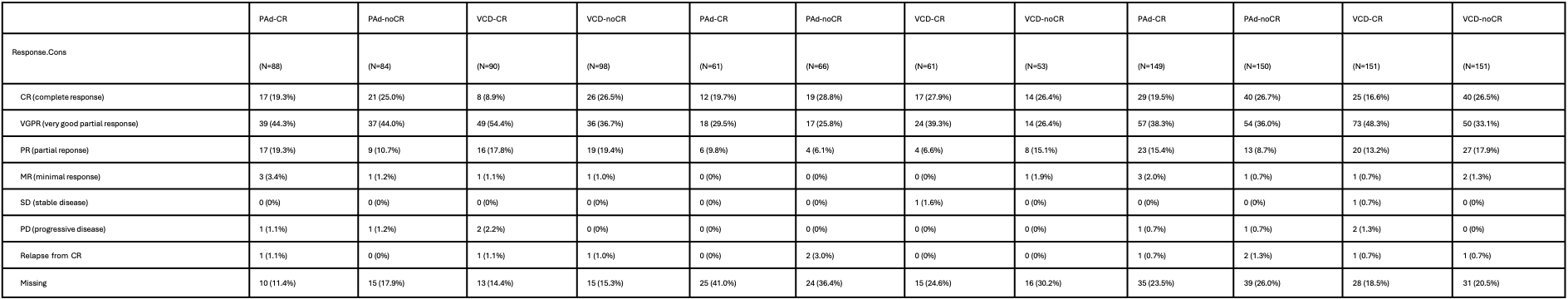


*Supplement Table 1: IMWG Response at end of consolidation, based on ITT of extended cohort (n=601 patients). Numbers/Proportions are listed separate for the subset of patients evaluable for hevylite-analysis and those, who are not*

| Covariate | Covariate levels | missing | Hazard Ratio | 95%-CI | p-value |
| --- | --- | --- | --- | --- | --- |
| HLCr norm | no | 0 | Ref |  |  |
|  | yes |  | 0.61 | [0.37;1.02] | 0.06 |
| HighRisk | no | 17 | Ref |  |  |
|  | yes |  | 1.67 | [1.00;2.80] | 0.051 |
| ISS | I | 0 | Ref |  |  |
|  | II |  | 1.66 | [0.94;2.95] | 0.08 |
|  | III |  | 2.49 | [1.20;5.13] | 0.01 |
| Age |  | 0 | 0.97 | [0.94;1.01] | 0.13 |

*Supplement Table 2: Multivariable Cox regression analysis to evaluate the prognostic impact of HLCr normalization on PFS in patients with continuous maintenance from the end of consolidation*

| Covariate | Covariate levels | missing | Hazard Ratio | 95%-CI | p-value |
| --- | --- | --- | --- | --- | --- |
| HLCr norm. | no | 0 | Ref |  |  |
|  | yes |  | 0.86 | [0.51;1.45] | 0.58 |
| HighRisk | no | 17 | Ref |  |  |
|  | yes |  | 1.87 | [1.10;3.20] | 0.02 |
| ISS | I | 0 | Ref |  |  |
|  | II |  | 1.04 | [0.57;1.88] | 0.90 |
|  | III |  | 1.84 | [1.01;3.33] | 0.046 |
| Age |  | 0 | 0.99 | [0.97;1.02] | 0.68 |

*Supplement Table 3: Multivariable Cox regression analysis to evaluate the prognostic impact of HLCr normalization on PFS in patients with response-adapted maintenance from the end of consolidation*

Supplement Figure 1:


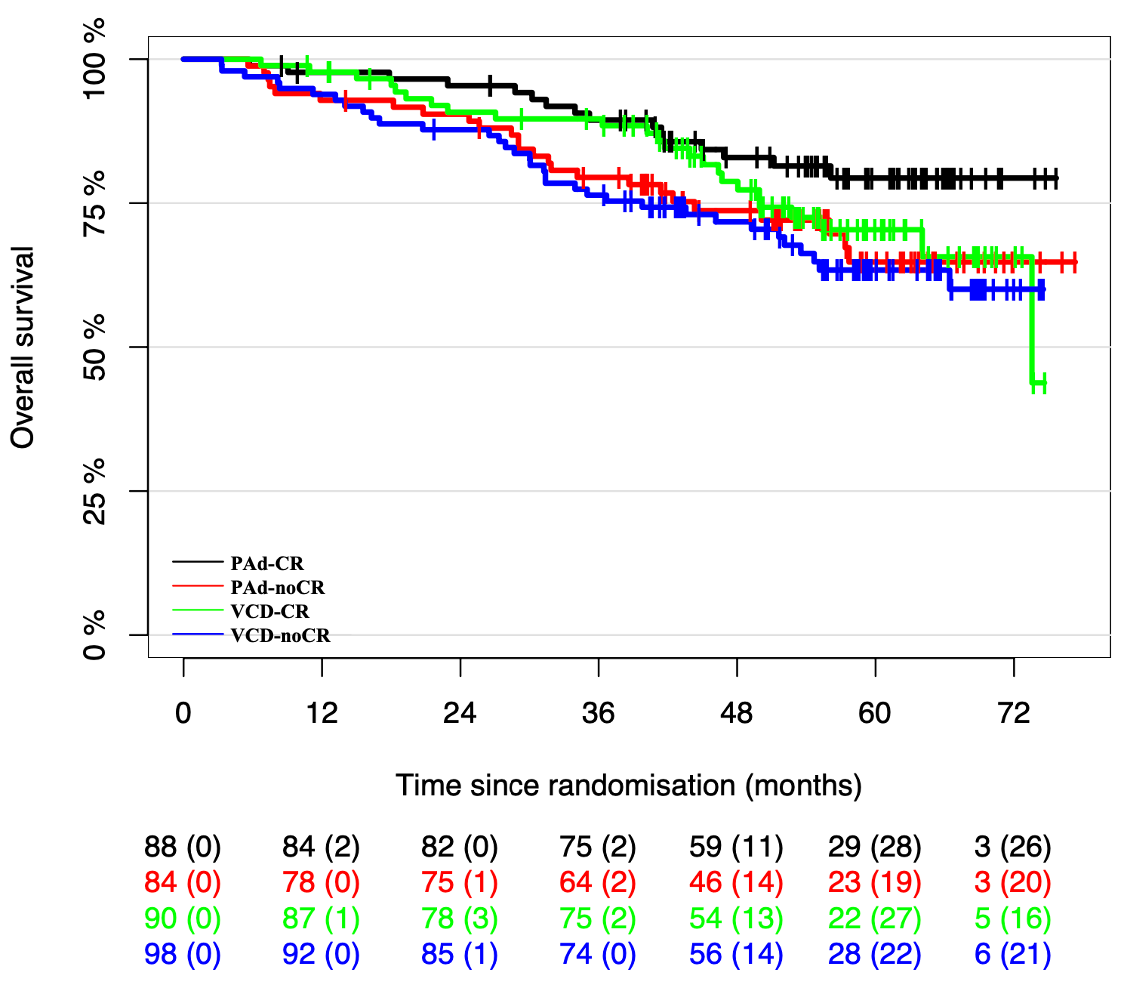


*Supplement Figure 1: With a median follow up of 57,6 months, 99 deaths were observed for the 360 evaluable patients. The distribution of OS for the subset of the Hevylite analysis, is calculated by the method of Kaplan and Meier, stratified by randomisation arm.*
